# Role of curcumin in oral infection and inflammation 

**DOI:** 10.3205/dgkh000575

**Published:** 2025-08-19

**Authors:** Karthik Shunmugavelu, Gautam Bhaskar

**Affiliations:** 1 Department of Dentistry, PSP Medical College Hospital and Research Institute Tambaram, Kanchipuram, Tamil Nadu, India; 2 Anatomy Department, Sree Balaji Medical College and Hospital, Bharath University, Chennai, Tamil Nadu, India

**Keywords:** curcumin, anti-inflammatory activity, antioxidant activity, antimicrobial activity, anticancer activity

## Abstract

Curcumin, which is a polyphenol from the rhizomes of *Curcuma longa*, has been found to possess anti-inflammatory, antioxidant, and antimicrobial activities. This systematic review examines the effectiveness of curcumin in treating oral inflammation, e.g., periodontal diseases, gingivitis, oral lichen planus (OLP), and radiation-induced oral mucositis (ROM). The studies in this review assess different curcumin formulations, including gels, hydrogels, nano-curcumin, and mouthwashes, as adjunctive agents in oral inflammatory diseases. The studies indicate that curcumin significantly decreases clinical markers of inflammation, improves healing, and reduces patient discomfort, warranting its use as an adjunctive therapeutic agent.

## Abstract (de)

Curcumin, ein Polyphenol aus den Rhizomen von *Curcuma longa*, hat sich als entzündungshemmend, antioxidativ und antimikrobiell erwiesen. Die vorliegende Übersicht untersucht die Wirksamkeit von Curcumin bei der Behandlung von Entzündungen im Mundraum, d. h. bei Parodontalerkrankungen, Gingivitis, oralem Lichen planus (OLP) und strahleninduzierter oraler Mukositis (ROM). Die Studien bewerten verschiedene Curcumin-Formulierungen, darunter Gele, Hydrogele, Nano-Curcumin und Mundspülungen, als ergänzende Wirkstoffe bei oralen Entzündungskrankheiten. Die Studien weisen darauf hin, dass Curcumin die klinischen Entzündungsmarker signifikant senkt, die Heilung verbessert und die Beschwerden der Patienten verringert, was seinen Einsatz als begleitendes therapeutisches Mittel rechtfertigt.

## Introduction

Oral inflammation includes periodontitis, gingivitis, oral lichen planus (OLP), and radiation-induced oral mucositis (ROM), each of which elicits pain, swelling, erythema, and compromised oral function, significantly impacting patients’ quality of life [1]. Periodontitis and gingivitis entail chronic inflammation of the periodontal tooth-supporting structures, which could result in loss of attachment and bone. OLP is a chronic T-cell–mediated autoimmune condition manifested by painful mucosal erythema, erosions, or ulcerations. ROM is an uncomfortable complication of radiotherapy in head and neck cancer patients, leading to oral mucosal inflammation and ulceration [2], [3].

These conditions result from complex interactions between microbial pathogens, host immune responses, and pro-inflammatory cytokines. Traditional treatments such as scaling and root planing (SRP), corticosteroids, chemical mouthwashes, and antibiotics aim to control inflammation and reduce microbial load, but may cause side effects such as antimicrobial resistance, taste disturbances, and mucosal atrophy. High recurrence rates highlight the need for new therapeutic agents [1].

Curcumin, a polyphenol from the *Curcuma longa* plant, has anti-inflammatory, antioxidant, antimicrobial, and anticancer activities based on inhibition of nuclear factor-kappa B (NF-κB), cyclooxygenase-2 (COX-2), lipoxygenase, and pro-inflammatory cytokines such as IL-1β, IL-6, and TNF-α. Its clinical application is, however, hampered by poor solubility and low bioavailability, [4]. To enhance its potency, formulations such as gels, hydrogels, nano-curcumin, and microemulsions have been synthesized for better stability, bioavailability, and drug targeting. The products are formulated as adjuncts to SRP in periodontal therapy and in mouthwashes to control oral biofilm and inflammation [5].

This review assesses curcumin’s therapeutic capability to treat oral inflammatory diseases based on clinical markers such as plaque index (PI), gingival index (GI), probing pocket depth (PPD), clinical attachment level (CAL), erythema, size of lesion, and pain. It further cites gaps in ongoing research where it is evident that curcumin may act as a very viable, natural alternative to modern conventional treatments against oral inflammation.

## Materials and methods

A literature search of studies was performed using the online databases PubMed, Scopus, and Cochrane Library. The review comprised randomized controlled trials, clinical studies, and meta-analyses assessing the effectiveness of curcumin in oral inflammation. Studies that measured clinical parameters such as plaque index (PI), gingival index (GI), probing pocket depth (PPD), clinical attachment level (CAL), erythema, lesion size, and pain were selected. The risk of bias was assessed with the Cochrane Collaboration’s tool for clinical trials.

## Results

The results of the studies included are summarized in Table 1 (Tab. 1), Table 2 (Tab. 2), Table 3 (Tab. 3), and Table 4 (Tab. 4) to give an overview of the effectiveness of various curcumin formulations in treating oral inflammatory conditions.

## Discussion

The present review depicts the therapeutic relevance of curcumin in the treatment of diverse inflammatory oral disorders such as periodontitis, gingivitis, OLP, and ROM. The observations consistently illustrate the anti-inflammatory, antioxidant, and antimicrobial functions of curcumin, attributing its applicability as a supplementary treatment. The efficacy of various formulations of curcumin, i.e., gels, hydrogels, nano-curcumin, and microemulsions, were discussed in the included studies. The clinical outcome variability can be explained by formulations, dosages, and drug delivery system variations. In particular, nano-curcumin preparations had higher bioavailability and therapeutic effects, with greater penetration and longer release.

In the management of periodontitis and gingivitis, most studies demonstrated significant improvements in clinical parameters such as PI, GI, PPD, and CAL, when curcumin was used as an adjunct to scaling and root planing (SRP) compared to SRP alone. Abdel-Fatah et al. [6] and Mohammad et al. [7] noted significant decreases in inflammatory biomarkers such as IL-1β, TNF-α, and salivary procalcitonin, indicating curcumin’s strong anti-inflammatory and antioxidant effects. In contrast, Malekzadeh et al. [8] did not find a change in PI but reported significant decreases in gingival inflammation and bleeding. This disparity may be due to variations in curcumin formulations, dosages, and study populations. A curcumin/zinc oxide (Cur/ZNP) hydrogel showed enhanced antimicrobial activity and improved alveolar bone preservation in an animal model, demonstrating the potential of hydrogels for localized drug delivery and sustained therapeutic effects [9].

For OLP, meta-analysis found that curcumin had no significant effect on erythema, lesion size, or overall pain. However, a subgroup analysis did show that a two-week treatment course significantly decreased pain, and thus the therapeutic efficacy of curcumin in OLP could be evaluated as duration- and frequency-dependent [10]. For ROM, Ramezani et al. [11] showed that curcumin mouthwash and nano-capsules both had strong effects on decreasing pain and severity, with 33% of patients still having no ulcers compared to the control group. This indicates that both topical and systemic curcumin treatment can be effective for ROM symptom control. Moreover, Rocha et al. [12] demonstrated that the combination of curcumin-based microemulsion mouthwash with photodynamic therapy (PDT) produced remarkable antimicrobial activity against *Candida albicans*, *Escherichia coli*, and methicillin-resistant *Staphylococcus aureus* biofilms, highlighting PDT’s potential as an adjunctive treatment to augment curcumin’s antimicrobial activity.

The differences in clinical effects among the studies can be explained by differences in curcumin preparations (gel, hydrogel, nano-capsules, and microemulsions), dosing, and drug delivery systems. Most of the nano-curcumin preparations had better bioavailability and therapeutic effects because of increased penetration and release over a period of time.

The mechanism of action of curcumin includes modulation of pro-inflammatory cytokines (IL-1β, IL-6, TNF-α) and inhibition of NF-κB and COX-2 signaling, suppressing oxidative stress and inflammation. Curcumin suppresses the expression of matrix metalloproteinases (MMPs) that participate in tissue damage and alveolar bone resorption in periodontal infections. It increases antioxidant enzyme activity, including superoxide dismutase (SOD) and catalase, which shields oral tissues from oxidative damage [5].

Additionally, curcumin’s antimicrobial properties are linked to its ability to disrupt microbial biofilms, inhibit bacterial growth, and interfere with bacterial quorum sensing, thereby reducing virulence factor production. Curcumin inhibits the growth of key periodontal pathogens, including *Porphyromonas gingivalis* and *Fusobacterium nucleatum*, by disrupting their cell membrane integrity and inhibiting protease activity. These multifaceted mechanisms contribute to its therapeutic effectiveness in managing oral inflammation [13].

### Clinical implications

The review highlights curcumin’s efficacy as a safe and effective adjunct treatment for the management of oral inflammation. Its use in gels and hydrogels improves clinical results in periodontitis and gingivitis. Nano-curcumin preparations provide enhanced bioavailability and patient compliance. While curcumin is promising in pain and inflammation relief in ROM, its efficacy in OLP is inconclusive.

### Integration into clinical practice 

The following options are promising:

- Adjunctive treatment of periodontitis and gingivitis: Curcumin hydrogels and gels may be utilized as useful adjuncts to conventional mechanical debridement, promoting clinical benefits.
- Potential in ROM management: Curcumin mouthwash and nano-capsules have potential in minimizing pain and severity of ROM, enhancing the quality of life of cancer patients receiving radiotherapy.
- Role in combination therapies: The combination of curcumin with new drug delivery systems, including hydrogels and photodynamic treatment, enhances its therapeutic effects to the fullest, providing a promising approach for the management of overall oral inflammation.

### Limitations and future directions 

While curcumin demonstrates promising findings, the heterogeneity of study designs, sample sizes, and treatment protocols makes it difficult to generalize the results, citing the necessity of long-term clinical trials with standardized formulations and dosages to define optimal therapeutic regimens. Future studies must address advanced drug delivery systems like hydrogels and nanoparticles to improve curcumin’s stability, bioavailability, and targeted delivery. Also, studying its synergistic actions with other anti-inflammatory drugs and probing its molecular mechanisms of action will further confirm its therapeutic potential.

## Conclusions

This review confirms curcumin’s potential as an adjunctive treatment for oral inflammatory conditions. Curcumin formulations, especially gels, hydrogels, and nano-capsules, enhance clinical outcomes in periodontitis, gingivitis, and ROM. Its role in OLP requires further exploration. Future research should focus on optimizing curcumin delivery systems and evaluating long-term clinical benefits.

## Notes

### Competing interests

The authors declare that they have no competing interests.

### Authors’ ORCIDs

- Shunmugavelu K: https://orcid.org/0000-0001-7562-8802
- Bhaskar G: https://orcid.org/0009-0000-8263-4768

### Funding

None. 

## Figures and Tables

**Table 1:**
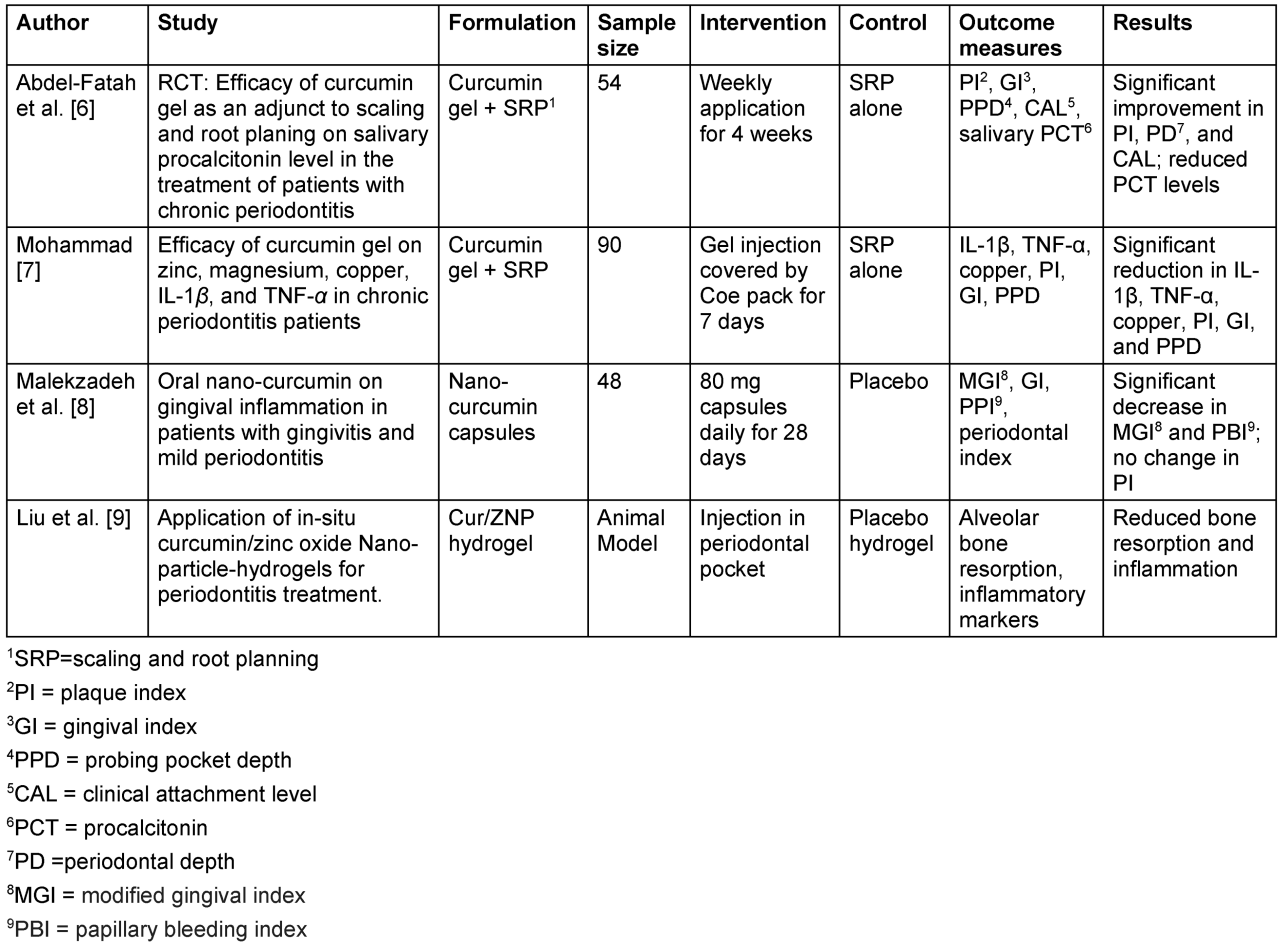
Curcumin in periodontitis and gingivitis

**Table 2:**
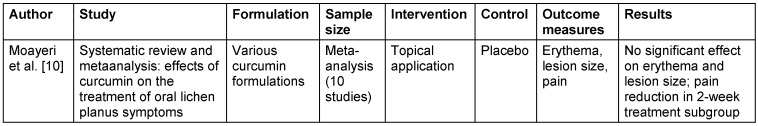
Curcumin in oral lichen planus (OLP)

**Table 3:**
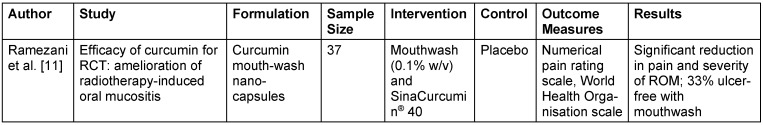
Curcumin in radiation-induced oral mucositis (ROM)

**Table 4:**
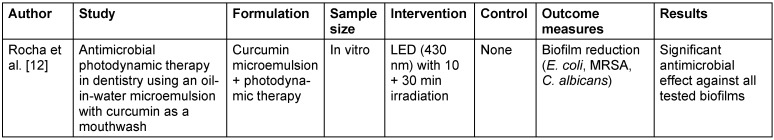
Photodynamic therapy and curcumin mouthwash

## References

[1] Idrees M, Kujan O (2024). Curcumin is effective in managing oral inflammation: An in vitro study. J Oral Pathol Med.

[2] Murakami S, Mealey BL, Mariotti A, Chapple ILC (2018). Dental plaque-induced gingival conditions. J Periodontol.

[3] Eversole LR (1997). Immunopathogenesis of oral lichen planus and recurrent aphthous stomatitis. Semin Cutan Med Surg.

[4] Idrees M, Kujan O (2023). A Curcumin-Based Oral Gel Has Potential Protective Efficacy against Oral Mucositis: In Vitro Study. J Pers Med.

[5] Peng Y, Ao M, Dong B, Jiang Y, Yu L, Chen Z, Hu C, Xu R (2021). Anti-Inflammatory Effects of Curcumin in the Inflammatory Diseases: Status, Limitations and Countermeasures. Drug Des Devel Ther.

[6] Abdel-Fatah R, Mowafey B, Baiomy A, Elmeadawy S (2023). Efficacy of curcumin gel as an adjunct to scaling and root planing on salivary procalcitonin level in the treatment of patients with chronic periodontitis: a randomized controlled clinical trial. BMC Oral Health.

[7] Mohammad CA (2020). Efficacy of Curcumin Gel on Zinc, Magnesium, Copper, IL-1, and TNF- in Chronic Periodontitis Patients. Biomed Res Int.

[8] Malekzadeh M, Kia SJ, Mashaei L, Moosavi MS (2021). Oral nano-curcumin on gingival inflammation in patients with gingivitis and mild periodontitis. Clin Exp Dent Res.

[9] Liu C, Chen Y, Bai H, Niu Y, Wu Y (2024). Characterization and application of in situ curcumin/ZNP hydrogels for periodontitis treatment. BMC Oral Health.

[10] Moayeri H, Rajabi A, Mohammadi M, Moghaddam SB (2024). Effects of Curcumin on the treatment of oral lichen planus symptoms: a systematic review and meta-analysis study. BMC Oral Health.

[11] Ramezani V, Ghadirian S, Shabani M, Boroumand MA, Daneshvar R, Saghafi F (2023). Efficacy of curcumin for amelioration of radiotherapy-induced oral mucositis: a preliminary randomized controlled clinical trial. BMC Cancer.

[12] Rocha MP, Ruela ALM, Rosa LP, Santos GPO, Rosa FCS (2020). Antimicrobial photodynamic therapy in dentistry using an oil-in-water microemulsion with curcumin as a mouthwash. Photodiagnosis Photodyn Ther.

[13] Hussain Y, Alam W, Ullah H, Dacrema M, Daglia M, Khan H, Arciola CR (2022). Antimicrobial Potential of Curcumin: Therapeutic Potential and Challenges to Clinical Applications. Antibiotics (Basel).

